# Identification of Immune-Related Prognostic mRNA and lncRNA in Patients with Hepatocellular Carcinoma

**DOI:** 10.1155/2022/5313149

**Published:** 2022-01-04

**Authors:** Dan Chen, Xiaoting Li, Hui Li, Kai Wang, Xianghua Tian

**Affiliations:** ^1^Department of Medical Engineering and Technology, Xinjiang Medical University, Urumqi 830011, China; ^2^Central Laboratory of Xinjiang Medical University, Urumqi 830011, China

## Abstract

**Background:**

As the most common hepatic malignancy, hepatocellular carcinoma (HCC) has a high incidence; therefore, in this paper, the immune-related genes were sought as biomarkers in liver cancer.

**Methods:**

In this study, a differential expression analysis of lncRNA and mRNA in The Cancer Genome Atlas (TCGA) dataset between the HCC group and the normal control group was performed. Enrichment analysis was used to screen immune-related differentially expressed genes. Cox regression analysis and survival analysis were used to determine prognostic genes of HCC, whose expression was detected by molecular experiments. Finally, important immune cells were identified by immune cell infiltration and detected by flow cytometry.

**Results:**

Compared with the normal group, 1613 differentially expressed mRNAs (DEmRs) and 1237 differentially expressed lncRNAs (DElncRs) were found in HCC. Among them, 143 immune-related DEmRs and 39 immune-related DElncRs were screened out. These genes were mainly related to MAPK cascade, PI3K-AKT signaling pathway, and TGF-beta. Through Cox regression analysis and survival analysis, MMP9, SPP1, HAGLR, LINC02202, and RP11-598F7.3 were finally determined as the potential diagnostic biomarkers for HCC. The gene expression was verified by RT-qPCR and western blot. In addition, CD4 + memory resting T cells and CD8 + T cells were identified as protective factors for overall survival of HCC, and they were found highly expressed in HCC through flow cytometry.

**Conclusion:**

The study explored the dysregulation mechanism and potential biomarkers of immune-related genes and further identified the influence of immune cells on the prognosis of HCC, providing a theoretical basis for the prognosis prediction and immunotherapy in HCC patients.

## 1. Introduction

Hepatocellular carcinoma (HCC), a primary liver cancer in hepatocytes, is a prevalent health problem and one of the most common malignant tumors [1]. According to 2018 statistics, HCC ranked sixth in incidence and fourth in mortality in the world, with about 840 000 new cases and more than 780 000 deaths annually [2]. Although the overall survival (OS) rate of patients with HCC has improved thanks to advances in surgical techniques, i.e., the 5-year survival rate being 18%, it is still very low [3]. Most patients with late HCC have a high rate of recurrence and metastasis after treatment, which may be one of the reasons for poor prognosis [4]. Poor clinical outcomes make it imperative that our understanding of HCC and early diagnosis and treatment approaches should be improved.

The occurrence of liver cancer is a complex process involving multiple risk factors, mainly hepatitis B and C infection, cirrhosis, excessive alcohol consumption, obesity, fatty liver, aflatoxin, and diabetes [5]. It is well known that hepatocellular carcinoma cells are extremely resistant to almost all conventional chemotherapeutics; therefore, so far, only a limited number of chemotherapeutics can be used to treat patients with hepatocellular carcinoma [6, 7]. Even after surgical resection or ablation, 70% of patients still have tumor recurrence within 5 years [8]. Once the tumor progresses to a late stage, the currently available drug therapy can only produce a small survival benefit, which is not cost effective [9]. In fact, most patients develop into an incurable late stage before discovered. Therefore, early detection and prevention of the development of liver cancer is the most effective strategy to improve the prognosis of patients.

The exact molecular mechanisms underlying the initiation and development of HCC remain unclear; however, it is known that the occurrence and development of liver cancer is associated with intrahepatic tumor microenvironment (TME) [10]. The HCC tumor microenvironment is a dynamic system composed of tumor cells, complex cytokine environment, extracellular matrix, immune cell subsets, etc. [11]. In this complex network, many immunosuppressive mechanisms, including aggregation of immunosuppressive cells, loss of antigen presentation, and activation of multiple signaling pathways favor immune tolerance and promote the progression of liver cancer [12, 13]. Immunotherapy for hepatocellular carcinoma is currently a research hotspot [14].

Long noncoding RNAs (lncRNAs) are RNA molecules with a length more than 200 bp [15], which can regulate gene expression and protein synthesis in many ways [16]. lncRNAs play an important role in the pathogenesis and development of human tumors, including liver cancer [17], by involving in the regulation of HCC proliferation, apoptotic migration, tumor genesis, and metastasis [18]. lncRNAs are divided into two categories: one is to promote cancer while the other is to inhibit the progression of tumors, both of which are equally important in cancer treatment [19].

Like other solid cancers, a large number of genetic alterations accumulate during the development of liver cancer. Therefore, this study screened differentially expressed genes of immune-related HCC by comparing with the control group to find potential diagnostic markers. Molecular mechanism of differentially expressed genes in hepatocellular carcinoma was identified by bioinformatics.

## 2. Materials and Methods

### 2.1. Data Collection and Difference Analysis

Gene expression profiles were collected from the 50 normal and 374 tumor liver tissues from The Cancer Genome Atlas (TCGA) database, which included mRNA and lncRNA expression profiles. The DESeq R software package was used to normalize the expression level of genes. The differentially expressed mRNA and differentially expressed lncRNA between the HCC and normal samples were analyzed using DESeq R software package. The |log2(fold change)| > 2 and *P* value < 0.05 were considered significant difference.

GSE149614 was obtained from the gene expression omnibus (GEO) database, which included gene expression profiles of 192 single synovial fibroblasts from 10 HCC patients and 8 no ntumor liver samples through single-cell RNA-seq (scRNA-seq) based on GPL24676.

### 2.2. Recognition of Immune-Related Genes

The immune-related DEmRs were screened out by intersecting the immune-related mRNAs in the ImmPort database with DEmRs, and the immune-related DElncRs were screened out by intersecting the immune-related lncRNAs in ImmLnc database with DElncRs.

### 2.3. Protein-Protein Interaction (PPI) Network

The immune-related DEmRs were put into Search Tool for the Retrieval of Interacting Genes (STRING) database (https://string-db.org.uk/), whose interaction score > 0.7 was screened to construct a PPI network and then visualized using Gephi software. The PPI network was imported into Cytoscape software to identify hub genes of the network by screening the degree of connectivity between genes.

### 2.4. Biological Function Analysis

The aforementioned ImmPort database was used to conduct biological process (BP) analysis by Gene Ontology (GO) and KEGG pathway enrichment analysis of immune-related DEmRs. The KEGG pathway of immune-related lncRNAs was analyzed with the ImmLnc database. *P* value < 0.05 was used as the cut-off criteria.

### 2.5. Clinical Significance for Hub Genes in HCC

OS analysis was performed with survival R software package. Cox regression analysis was used to identify the impact of key genes on the prognosis of HCC patients. The area under the receiver operating characteristic (ROC) curve was used to assess the potential diagnostic accuracy for abnormal expression of selected genes.

### 2.6. Processing of the scRNA-Seq Data

For GSE149614, the filter criteria were 300 < nFeature_RNA < 3000 and percent. The visualization of unsupervised clustering was performed using the t-distributed stochastic neighbor embedding (tSNE) method. Different cell clusters were annotated by the SingleR package.

### 2.7. Subjects

A total of ten HCC and ten normal whole blood samples and liver tissue samples were obtained from the patient admitted to the First Affiliated Hospital of Xinjiang Medical University. Patients had signed the informed consent. This study was approved by the medical ethics committee of the First Affiliated Hospital of Xinjiang Medical University (No. K202010-06).

### 2.8. RNA Extraction and RT-qPCR

The total RNA of the tissue samples was extracted by trizol (TaKaRa, Dalian, China) from the HCC and normal groups and reversely transcribed into complementary DNA (cDNA) using the PrimeScript RT reagent kit (TaKaRa, Dalian, China) according to the manufacturer's protocol. Real-time quantitative PCR (RT-qPCR) was performed using SYBR GreenPCR kit (Thermo Fisher Scientific, Massachusetts, USA). The expression of genes was normalized with GAPDH and calculated using 2^−ΔΔCt^ method. The primers of this study are shown in Table 1.

### 2.9. Western Blot

The protein expression levels of the hub mRNAs were verified using the western blot (WB) method. The liver tissue samples were lysed on ice in the presence of RIPA buffer. The proteins were quantified using BCA protein assay kit (Beyotime, Shanghai, China) and separated by SDS-PAGE and subsequently transferred to PVDF membranes, which were incubated overnight with primary antibodies (Abcam, Massachusetts, USA). After incubation with secondary antibodies (Abcam, Massachusetts, USA), protein bands were visualized using Chemi Imaging (Vilber Lourmat, France). GAPDH was used as the internal reference protein.

### 2.10. Flow Cytometry

Lymphocyte separation solution (TBD, Tianjin, China) was used to extract lymphocytes from blood samples. Cells were surface-labeled with anti-CD4-FITC (BD, California, USA), anti-CD8-PE antibody (BD, California, USA), anti-CD45RO-PE (BD, California, USA), or anti-CD38-PC5.5 (BD, California, USA) at 4 °C. The results of detection were analyzed using Kaluza v2.1.1 software.

### 2.11. Statistical Analysis

Statistical analyses were performed using the SPSS 21.0 software (IBM, NY, USA). Measurement data were expressed as mean ± standard deviation. The significance of the different expressions between groups was tested using Student's *t*-test. The correlation analysis between immuno-related RNAs and immune cells was tested using Pearson correlation analysis. Inspection level *α* = 0.05 and *P* < 0.05 was indicative of statistical significance.

## 3. Results

### 3.1. Differentially Expressed Genes in HCC

**Figure 1:**
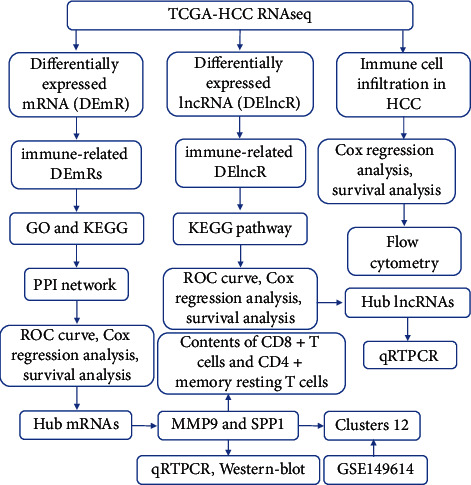
Flow chart of this study.

**Figure 2:**
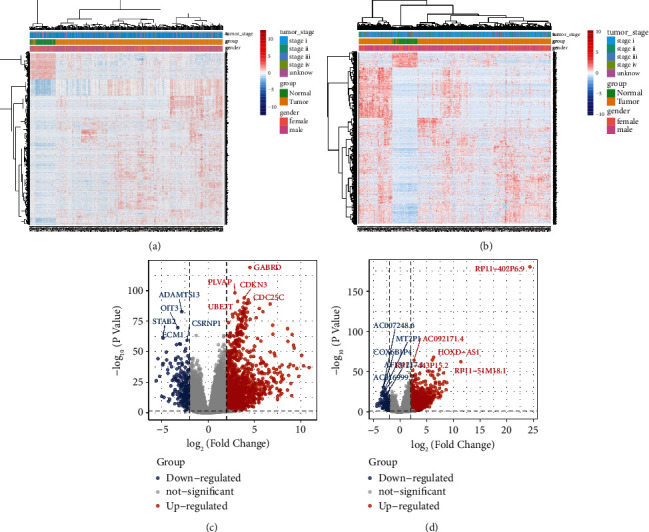
Identification of differentially expressed genes. (a) Heatmap of differentially expressed mRNAs between the HCC and control groups. (b) Heatmap of differentially expressed lncRNAs between the HCC and control groups. (c) Volcano map of differentially expressed mRNAs between the HCC and control groups. (d) Volcano map of differentially expressed mRNAs between the HCC and control groups.

The flow chart of this study is shown in Figure 1. By comparing the difference in the TCGA database between the HCC and normal control group, 1613 differentially expressed mRNAs and 1237 differentially expressed lncRNAs (Figures 2(a) and 2(b)) were obtained. 1368 upregulated genes and 245 downregulated genes (Figure 2(c)) were found in DEmRs while 1129 upregulated genes and 108 downregulated genes were found in DElncRs (Figure 2(d)).

### 3.2. Immune-Related Differentially Expressed mRNAs

**Figure 3:**
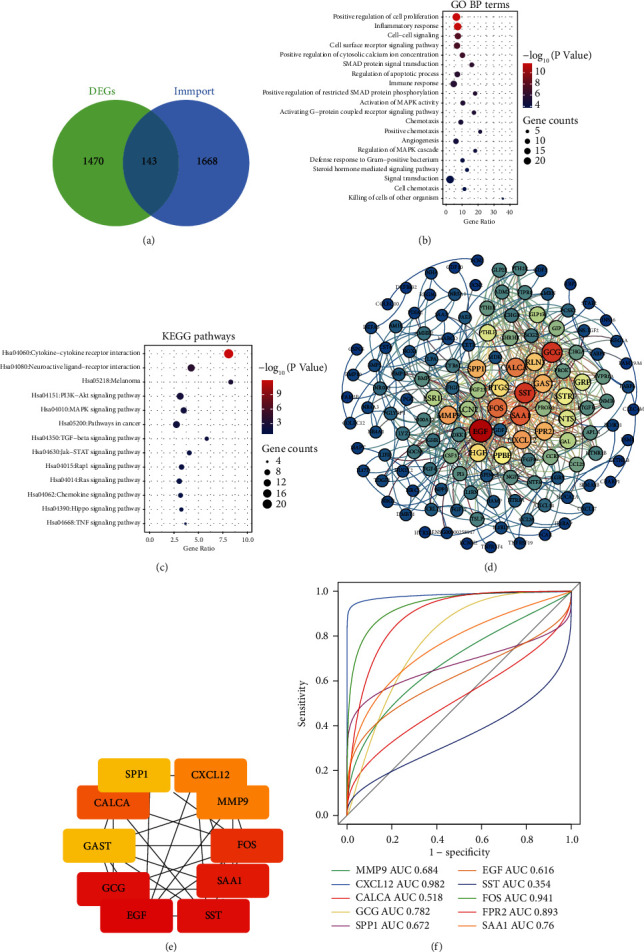
Identification of differentially expressed immune-related mRNAs. (a) The intersection of differentially expressed mRNAs and immune-related genes in the ImmPort database. (b) The main biological process of immune-related DEmR enrichment. (c) The main KEGG pathway of immune-related DEmR enrichment. (d) The PPI network of immune-related DEmR. (e) The gene network of the top 10 degrees in the PPI network. (f) ROC curve of hub genes.

Comparing differentially expressed mRNAs with immune-related genes in the Immport database, 143 immune-related DEmRs (Figure 3(a)) were identified. Enrichment analysis suggested that these genes were mainly involved in the positive regulation of cell proliferation, inflammatory response, and activation of MAPK biological process (BP) (Figure 3(b)) and also enriched in KEGG signaling pathways such as cytokine receptor interaction, PI3K-AKT signaling pathway, and MAPK signaling pathway (Figure 3(c)). These immune-related DEmRs were subjected to PPI network analysis to identify their interaction relationship (Figure 3(d)), and the top 10 genes with the largest degree of connectivity in the network were screened and identified as hub mRNAs (EGF, SST, GCG, SAA1, FOS, CALCA, MMP9, CXCL12, FPR2, and SPP1) (Figure 3(e)). ROC curves showed that the AUC values of EGF, GCG, SAA1, FOS, MMP9, CXCL12, FPR2, and SPP1 were greater than 0.6 (Figure 3(f)).

### 3.3. Identification of Key DEmRs

**Figure 4:**
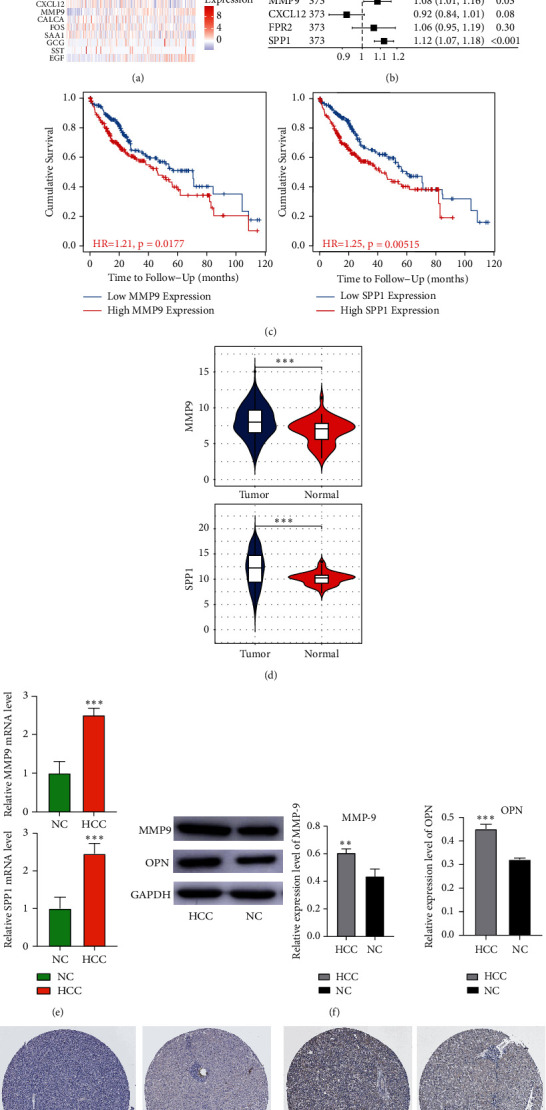
Key DEmRs affecting the prognosis of HCC. (a) The expression of key DEmRs and the HCC risk score. (b) The prognostic risk of key DEmRs in HCC analyzed by Cox regression. (c) Survival analysis of key DEmRs. (d) The expression of MMP9 and SPP1 in TCGA database between the HCC and control group. (e) The mRNA levels of MMP9 and SPP1 were detected by RT-qPCR in liver tissues from the HCC and NC groups. (f) The protein expression of MMP9 and OPN was detected by western blot in liver tissues from the HCC and NC groups. Immunohistochemical detection results of MMP9 (g) and SPP1 (h) in HCC and NC groups in The Human Protein Atlas database. NC: normal control and HCC: hepatocellular carcinoma. ^∗∗^*P* < 0.01, ^∗∗∗^*P* < 0.001.

Ten hub genes were analyzed for the risk score, and their expression changes were associated with the prognostic risk of HCC (Figure 4(a)). Cox regression analysis found that EGF, GCG, MMP9, and SPP1 were all risk factors for HCC (Figure 4(b)). Importantly, low expression of MMP9 and SPP1 significantly improved the overall survival time of HCC (Figure 4(c)), whose expression in HCC patients in TCGA data were high (Figure 4(d)). This was also confirmed by the results of RT-qPCR and western blot experiments (Figures 4(e) and 4(f)). Using the immunohistochemical results from The Human Protein Atlas database (https://www.proteinatlas.org/), it was shown that the expression of MMP9 as well as SPP1 was significantly higher in HCC patients than that in controls (Figures 4(g) and 4(h)).

**Figure 5:**
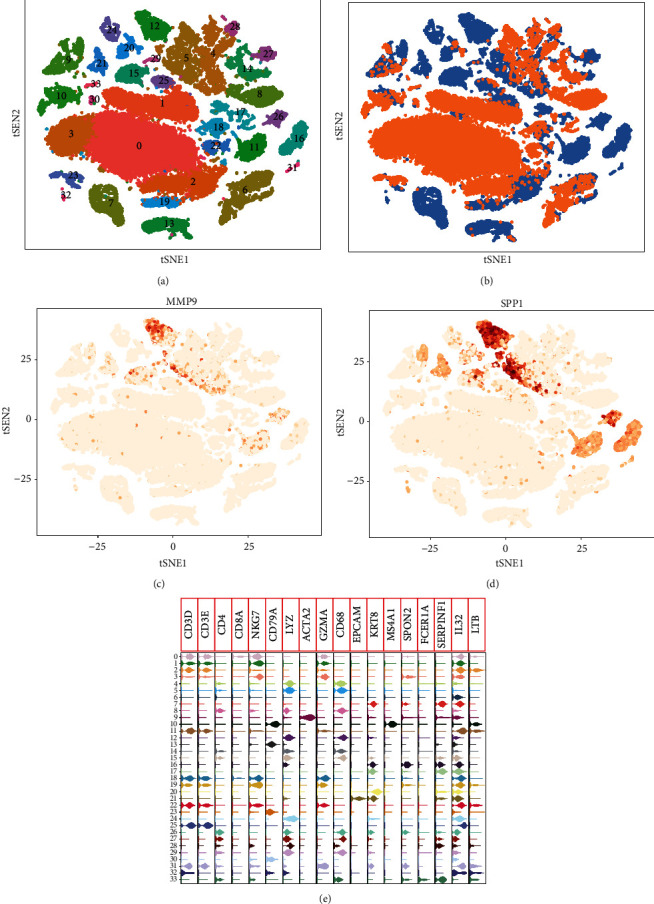
Key DEmRs expression in immune cells. (a) The tSNE algorithm clustered cells into 33 clusters. (b) The cell clusters were matched to the sample types for HCC or control. Blue is HCC and orange is control. (c) MMP9 mainly expressed in cluster 12. (d) SPP1 mainly expressed in cluster 12. (e) The expression of immune markers in all clusters of cells.

In addition, according to the results of GSE149614, we clustered cells into 33 separate clusters (Figure 5(a)). Through comparing with the clinical phenotypes, we found that different clusters matched HCC or control groups (Figure 5(b)). Interestingly, we found that MMP9 and SPP1 were mainly expressed in cluster 12, corresponding to the HCC group (Figures 5(c) and 5(d)). Cluster 12 was annotated as macrophage (Figure 5(e)).

### 3.4. Immune-Related Differentially Expressed lncRNAs

**Figure 6:**
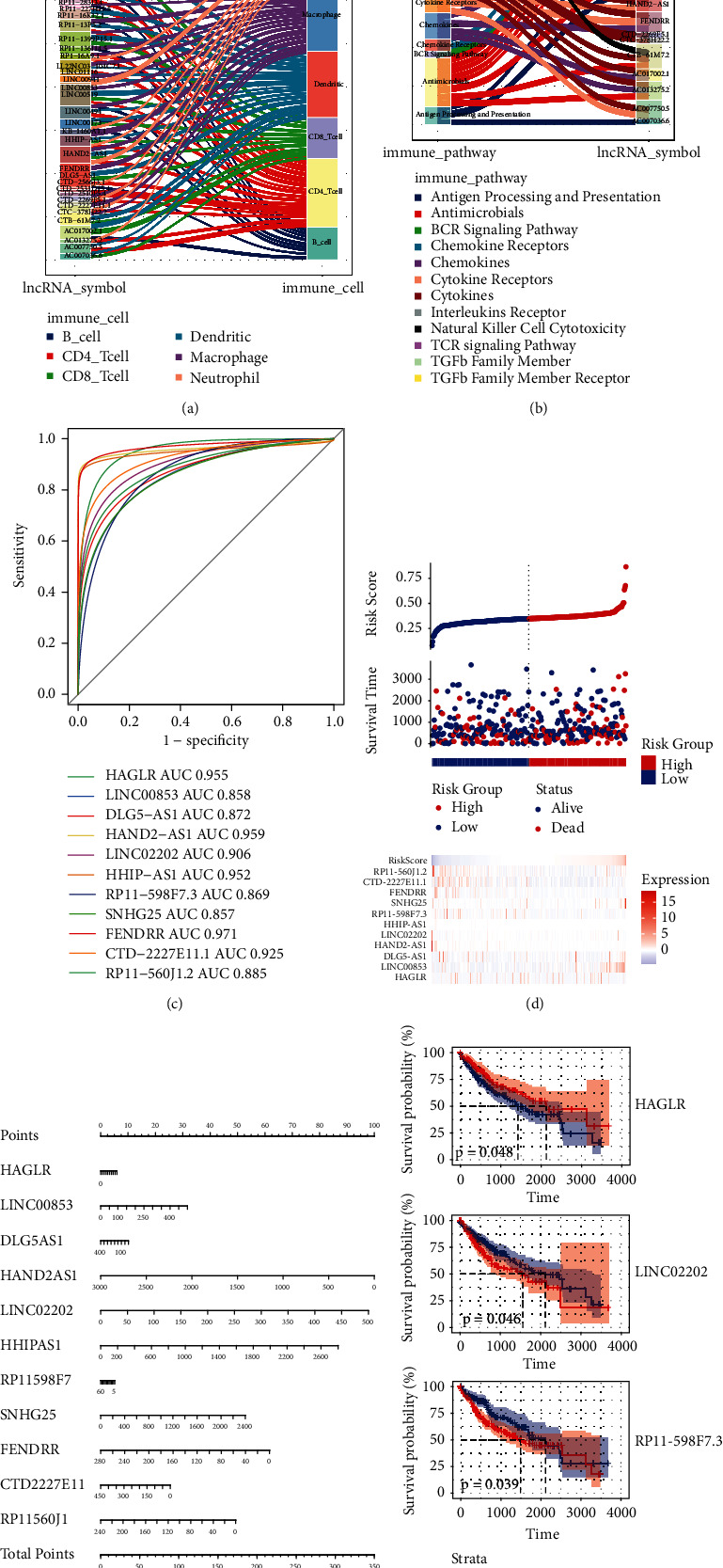
Identification of key immune-related DElncRs. (a) Screening of immune-related DElncRs. (b) KEGG signaling pathway of immune-related DElncR enrichment. (c) ROC curve of core DElncRs. (d) The expression of core DElncRs and the HCC risk score. (e) Nomogram of core genes for HCC. (f) Survival analysis of core genes.

Furthermore, by comparing with the ImmLnc database, 39 immune-related DElncRs (Figure 6(a)) were identified, which were associated with B cell, CD4+ T cell, CD8+ T cell, dendritic cell, macrophage, and neutrophil correlations and also significantly involved in the KEGG signaling pathway related to immune inflammation, such as TGF-beta family member, TCR signaling pathway, and interleukins receptor (Figure 6(b)). Through ROC curves of 39 DElncRs, 11 DElncRs with AUC values greater than 0.85 were identified as hub lncRNAs (Figure 6(c)). The results of risk score analysis showed that the expression changes of 11 DElncRs affected the prognosis of HCC (Figure 6(d)). Cox regression analysis was performed on 11 DElncRs, and their impact on the prognosis of HCC was demonstrated by a nomogram (Figure 6(e)). Low expression of HAND2AS1 and FENDRR and high expression of LINC02202 and HHIPAS1 were prognostic risk factors for HCC. In addition, HAGLR, LINC02202, and RP11-598F7.3 had a significant impact on the survival of HCC patients (Figure 6(f)).

**Figure 7:**
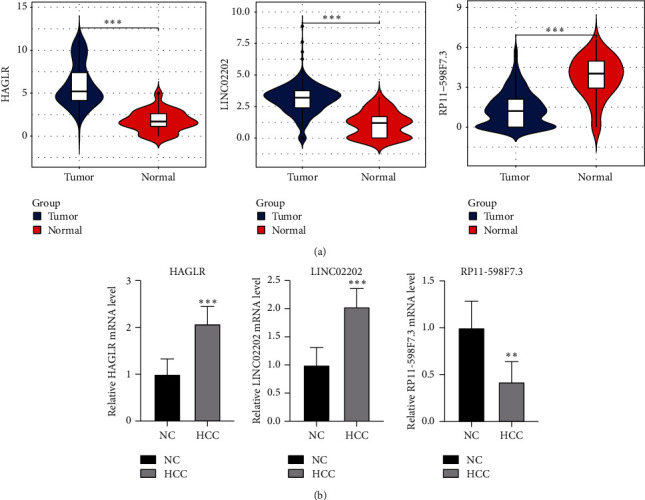
The expression of key lncRNAs in the TCGA Database. (a) The expression of key lncRNAs in the TCGA Database. (b) The expression of core genes in the HCC and NC groups detected by RT-qPCR. NC: normal control and HCC: hepatocellular carcinoma. ^∗∗^*P* < 0.01, ^∗∗∗^*P* < 0.001.

In the TCGA database, it was found that HAGLR and LINC02202 were upregulated and RP11-598F7.3 was downregulated compared with the control group (Figure 7(a)). The results of RT-qPCR experiments verified that HAGLR and LINC02202 were highly expressed in HCC while RP11-598F7.3 was lowly expressed (Figure 7(b)).

### 3.5. Immune Cell Infiltration in HCC

**Figure 8:**
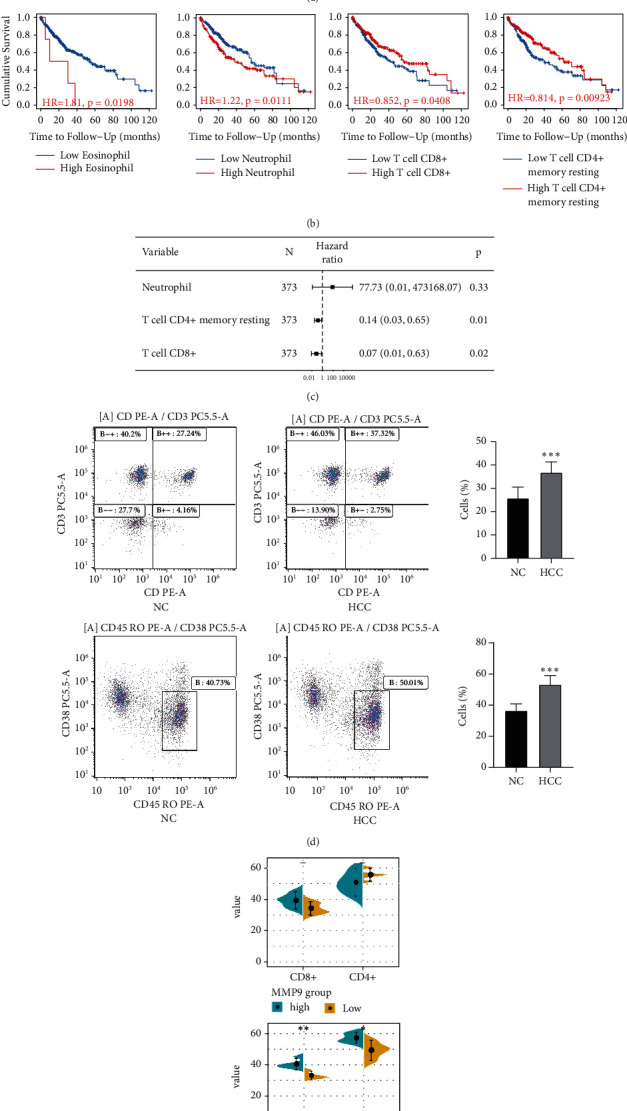
The effect of immune cells on the prognosis of HCC patients. (a) The infiltration level of immune cells in the HCC and NC groups. (b) Survival analysis of immune-infiltrating cells. (c) Forest map's effect of immune cells on the prognosis of liver cancer. (d) CD8 + T cells and CD4 + memory resting T cells in the HCC and NC groups were detected by flow cytometry. NC: normal control and HCC: hepatocellular carcinoma. ^∗∗∗^*P* < 0.001. (e) The contents of CD8 + T cells and CD4 + memory resting T cells in peripheral blood of HCC patients with high expression of MMP9 or SPP1 compared to those with the low expression. ^∗^*P* < 0.05, ^∗∗^*P* < 0.01.

To further identify the role of immune cells in HCC, the infiltration levels of 22 immune cells were analyzed using CIBERSORT (Figure 8(a)). Survival analysis showed that low expression of eosinophil and neutrophil, high expression of CD4+ memory resting T cells and CD8+ T cells could significantly improve the OS of HCC patients (Figure 8(b)). Cox regression analysis suggested that CD4+ memory resting T cells and CD8+ T cells were prognostic protective factors for HCC (Figure 8(c)). In addition, through examining the differences between eosinophil, neutrophil, CD4+ memory resting T cells, and CD8+ T cells in the blood of patients in the HCC and control groups by flow cytometry (Figure 8(d)), the difference in the levels of CD4 + memory T cells and CD8+ T cells between the HCC and normal groups were confirmed. The statistical results showed that the CD8+ T cells and CD4 + memory resting T cells content in HCC was significantly higher than that in the control group.

Moreover, we divided HCC samples into MMP9 or SPP1 high expression (5 samples) and low expression groups (5 samples), respectively. The contents of CD8 + T cells and CD4 + memory resting T cells in the HCC samples with high expression of SPP1 were higher than those with low expression of SPP1 (Figure 8(e)). However, the difference was not significant for the contents of CD8 + T cells and CD4 + memory resting T cells in the HCC samples with high expression of MMP9 as they was higher than those with low expression of MMP9. The results of correlation analysis showed that CD8 + T cells and CD4 + memory resting T cells were positively correlated with MMP9, SPP1, HAGLR, and LINC02202, while there was a negative correlation with RP11-598F7.3 (Figure S1).

## 4. Discussion

The immunotherapy is a promising but complicated treatment strategy since the liver itself is also an immune organ, which can enhance or inhibit the immune response of tumors generated in vivo [20]. It was found that about 25% of HCC were classified as “immune-specific class” according to gene expression profiles [21]. This study identified MMP9 and SPP1 as key DEmRs which were associated with immune in HCC. The immune cell infiltration had found that differences in infiltration of CD4+ memory resting T cells and CD8+ T cells may affect the prognosis of HCC. MMP9 and SPP1 were highly expressed in the macrophage of HCC patients and had positive correction with CD4+ memory resting T cells and CD8+ T cells. These results strengthen the association between immune cells and immune-related genes.

The immune-related genes screened were involved in a large number of biological functions and signaling pathways related to the occurrence and development of HCC. Cell proliferation and apoptosis have always been the molecular indicators for evaluating the development and progression of hepatocellular carcinoma [22, 23]. T cell receptors can mediate antigen-dependent tumor cytotoxicity, directly induce cell death through membrane-bound Fas ligand, and inhibit tumor proliferation through secretion of IFN-gamma [24]. The PD-1/PD-L1 pathway also inhibits the survival and growth of T cells in HCC by inhibiting T cell receptor signaling [14]. Several components of the MAPK cascade and PI3K-AKT signaling pathway are promising targets in HCC [25]. The final effects of the MAPK pathway are ERK 1/2, JNK, and p38. The expression of ERK 1/2 and JNK is upregulated, leading to the transcription of genes related to cell proliferation, survival, differentiation, and migration [26]. Induction of apoptosis, inhibition of cell proliferation, migration, and invasion by inhibiting the PI3K-AKT pathway is a therapeutic target for the treatment of HCC [27, 28]. TGF-*β* is also a common therapeutic target for the treatment of HCC [29] since serum TGF-*β* level is elevated in HCC patients and has been a long-term biomarker for HCC [30]. AKT signal transduction is thought to promote tumor formation by inhibiting TGF-*β*-induced apoptosis, which in turn activates Wnt/*β*-catenin signaling, thus further promoting the occurrence of HCC [15]. Inflammatory cells promote the occurrence of HCC by releasing ROS, RNS, lipid peroxidation, and abnormal expression of cytotoxic cytokines [31]. The complex interaction of different proinflammatory factors (such as interleukin-6 or TNF-*α*) with anti-inflammatory cytokines (TGF-*α* and TGF-*β*) and their signaling pathways is involved in the occurrence and development of liver cancer [32].

Multivariate analysis confirmed that the expression of matrix metalloproteinase 9 (MMP9) was an independent predictor of OS in hepatocellular carcinoma [33], which is consistent with our results. A large number of studies have confirmed that the high expression of MMP9 in HCC promotes the proliferation, migration, and invasion of HCC cells [34, 35]. MMP9 is particularly important for tumor invasion and metastasis in that it can degrade ColIV [36]. Secreted phosphoprotein 1 (SPP1) has been shown to be upregulated in HCC patients with a poor prognosis [37]. Osteopontin (OPN; gene SPP1) is also associated with cirrhosis cancer risk [38]. It has been recognized as a potential marker of early recurrence and poor prognosis as well as a metastasis-related gene of HCC [39].

Differential expression of HAGLR was upregulated in HCC [40, 41]. Mounting evidence suggests that high expression of HAGLR is related to clinicopathological features such as tumor size, lymph node metastasis, differentiation, TNM stage, and prognosis [42, 43]. LINC02202 can regulate the expression of PIK3R1 and FOXO1 genes in PI3K signaling pathway [44]. RP11-598F7.3 was found to be connected with Wilms tumor in the tumor stage and histological grade [45]. There are few studies on the direct relationship between LINC02202, RP11-598F7.3, and HCC, but our results suggested that LINC02202 and RP11-598F7.3 were prognostic risk factors for HCC.

Furthermore, the study found that CD4+ memory resting T cells and CD8+ T cells were protective factors for HCC. The CD8+ T lymphocytes and CD4+ memory T cells significantly increased in HCC [46]. In the elimination phase of HCC, emerging cancer cells can be recognized and killed by many immune cells, such as CD8+ and CD4+ T cells [47]. CD4+ T lymphocytes have been reported to inhibit the development of liver cancer and mediate tumor regression [48]. An increase in the percentage of cytotoxic CD4+ T cells was associated with a strong prognosis in both disease-free survival and OS [49]. CD8+ T cells are an important subset of T cells with antitumor activity mediated by the release of cytotoxic molecules, whose role in determining clinical efficacy in many cancers is obvious [50]. Moreover, CD4+ memory resting T cells and CD8+ T cells were all increased in HCC with high expression of the SPP1 group. Zheng et al. found that high expression of SPP1 may be involved in regulating the polarization of macrophages toward M2 phenotype and reducing CD8 + T cells [51]. There also is a study showing that SPP1 may contribute to tumor progression by promoting immune cell infiltration [52]. Further expanded samples for clinical studies and basic experiments are also needed to validate our results.

Therefore, the role of immune-related molecular therapy in HCC patients is indisputable, and the results of this study may provide a new direction for the diagnosis and treatment of HCC. However, this study also has some limitations. Our analytical data came from public databases, and the sample size used for experimental validation of the important results of this study was small. Subsequent cell culture and animal models are also needed to further investigate the underlying mechanisms of HCC. In addition, the application potential of the key markers identified in the study needs further validation.

## 5. Conclusion

In this study, MMP9, SPP1, HAGLR, LINC02202, and RP11-598F7.3 are considered as potential diagnostic biomarkers for hepatocellular carcinoma, and CD4+ memory resting T cells and CD8+ T cells are believed to be involved in HCC immune control. These results are expected to provide a new perspective for the molecular level interventional research and treatment of liver cancer.

## Figures and Tables

**Table 1:** The primers for qRT-PCR.

Genes	Primers
GAPDH	F: 5′-TGAAGGTCGGAGTCAACGGATTT-3′
	R: 5′-GCCATGGAATTTGCCATGGGTGG-3′
MMP9	F: 5′-GGGACGCAGACATCGTCATC-3′
	R: 5′-TCGTCATCGTCGAAATGGGC-3′
SPP1	F: 5′-TCACCTGTCATACCAGTT-3′
	R: 5′-TGGGTTG-3′
HAGLR	F: 5′-GATCCCCACCTTCCCCAAAG-3′
	R: 5′-TCTCCGACTGAGGTTTGCAC-3′
LINC02202	F: 5′-AACCAAGAGCGAAGCCAAGA-3′
	R: 5′-GCTTGGACACAGACCCTAGC-3′
RP11-598F7.3	F: 5′-CAGGACTACCGAGCACCAGGAC-3′
	R: 5′-TGACTCTCCTCAGCCAGCATCG-3′

## Data Availability

The data used to support the findings of this study are available from the corresponding author upon request.

## Supplementary Materials

Supplementary Materials Figure S1: The correlation between genes and immune cells. Red denotes positive correlation, and blue denotes negative correlation. ^∗∗^*P* < 0.01, ^∗∗∗^*P* < 0.01.
