# Supplementary figures and images for: Identification of Immune-Related Prognostic mRNA and lncRNA in Patients with Hepatocellular Carcinoma
Source: J Oncol. 2022 Jan 4;2022:5313149. doi: 10.1155/2022/5313149 (PMC8752260; doi:10.1155/2022/5313149)

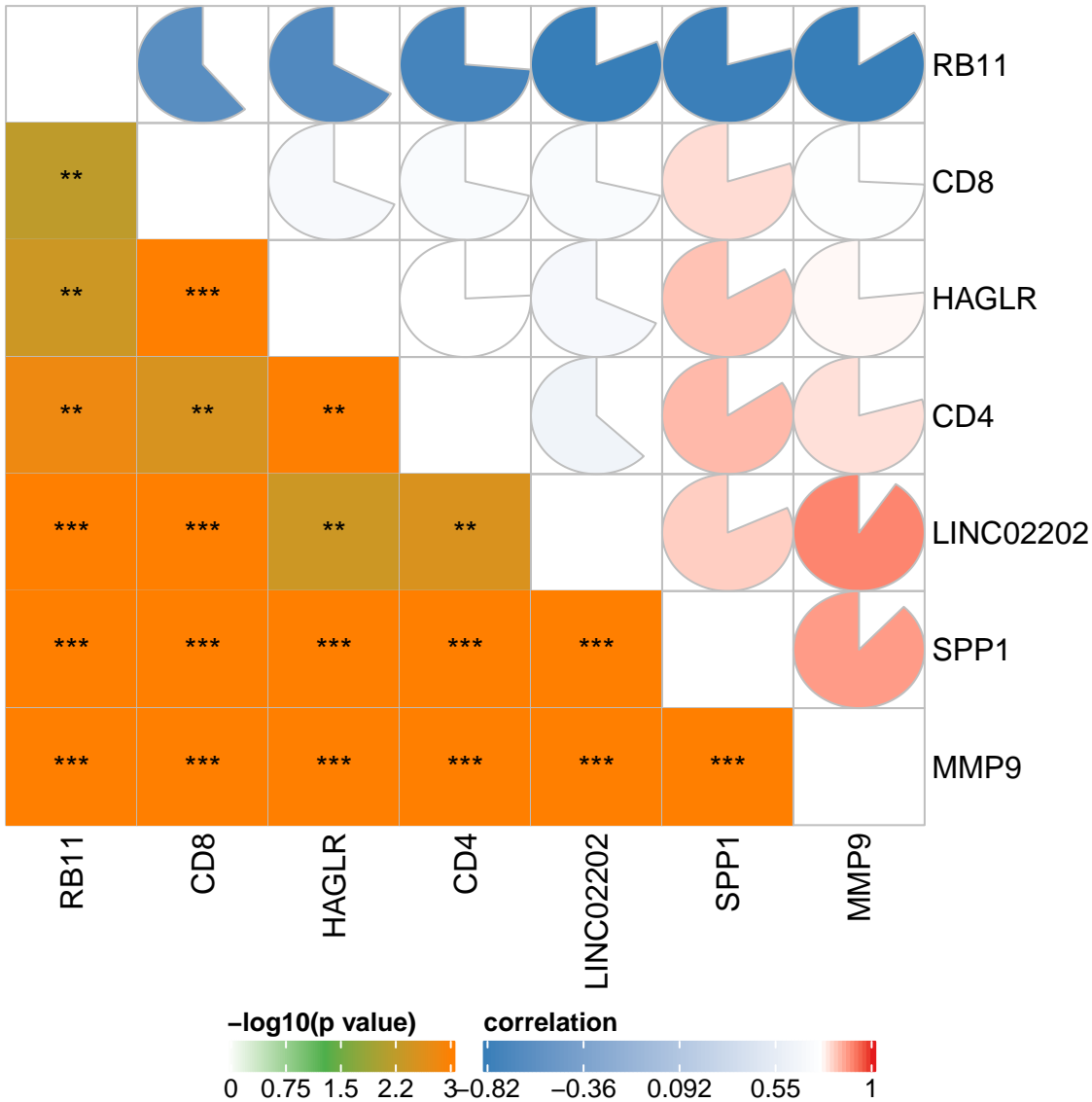

Supplement: Supplementary Materials — Figure S1: The correlation between genes and immune cells. Red denotes positive correlation, and blue denotes negative correlation. ∗∗P < 0.01, ∗∗∗P < 0.01. [file 5313149.f1.pdf]
